# Correction: Automated self-service cohort selection for large-scale population sciences and observational research: The California Teachers Study researcher platform

**DOI:** 10.1371/journal.pone.0347515

**Published:** 2026-04-21

**Authors:** The PLOS One Staff

## Notice of Republication

This article was republished on April 6, 2026, to correct an error in the article title. The words “Teachers Study” are miscapitalized in the article title. The correct title is: Automated self-service cohort selection for large-scale population sciences and observational research: The California Teachers Study researcher platform. The correct citation is: Lacey JV Jr, Spielfogel ES, Benbow JL, Savage KE, Lin K, Anderson CAM, et al. (2025) Automated self-service cohort selection for large-scale population sciences and observational research: The California Teachers Study researcher platform. PLoS One 20(5): e0296611. https://doi.org/10.1371/journal.pone.0296611. The publisher apologizes for the error. Please download this article again to view the correct version. The originally published, uncorrected article and the republished, corrected articles are provided here for reference.

## Supporting information

S1 File Originally published, uncorrected article. (PDF)

S2 File Republished, corrected article. (PDF)

## References

[1] Lacey JV Jr, Spielfogel ES, Benbow JL, Savage KE, Lin K, Anderson CAM, et al. Automated self-service cohort selection for large-scale population sciences and observational research: The California Teachers Study researcher platform. PLoS One. 2025;20(5):e0296611. doi: 10.1371/journal.pone.0296611 40354347 PMC12068635

